# Resection of the entire first rib for giant osteochondroma by trans manubrial approach: a case report and review of the literature

**DOI:** 10.1186/s13019-024-02902-9

**Published:** 2024-06-24

**Authors:** Yong Chen, Kun Deng, Chang Zhao, Wei Xiao, Zhong Tang

**Affiliations:** Department of Cardiothoracic Surgery, Second People’s Hospital of Neijiang, Neijiang, 64100 Sichuan Neijiang China

**Keywords:** Giant osteochondroma, First rib tumor, Inverse L-shaped incision, Trans manubrial approach, Resection

## Abstract

**Background:**

First rib tumors are extremely rare. Its compression of neurovascularity can easily lead to severe complications such as thoracic outlet syndrome, so early surgical resection is crucial. However, there is no standardized approach to surgery.

**Case presentation:**

A previously healthy 18-year-old Chinese male undergoes a chest computed tomography (CT) scan that incidentally reveals a raised calcified mass on the right first rib, which is most likely an osteochondroma when combined with magnetic resonance imaging (MRI). We achieved excellent results with resection and thoracic reconstruction by adopting an inverse L-shaped incision in the anterior chest and a longitudinal split of the sternum.

**Conclusions:**

Our practice provides great reference for the surgical management of first rib tumors.

## Background

Tumors on the ribs are uncommon, with reported incidents of bone tumors ranging from 3 to 8%. Osteochondroma of bone is a rare tumor in the ribs, accounting for about 8% of rib tumors, which is scarce to be present in the first rib [1]. Giant osteochondromas in the first rib pose challenges for surgical removal due to difficulties in exposure and the vulnerability of neurovascular structures. Here, we presented a case of successful resection of a giant osteochondroma on the first rib by using an inverse L-shaped incision in the manubrial part of the sternum and completed the reconstruction plate. Additionally, we conducted a comprehensive review of all reported cases of osteochondroma of the first rib, as summarized in Table 1.

**Table 1:** Literature review of osteochondroma of the first rib

Reference/time	Sex	Age	Side	Symptom	Tumor size(cm)	Occupying site	Approach
K. Bhaskaranand 1997 [1]	F	19	L	clavicle was protuberant	ND	Anterior	anterior approach
K. Bhaskaranand 1997 [1]	F	14	L	radicular pain along the left upper limb	ND	Anterior	anterior approach
O’Brien et al. 2011 [2]	F	12	R	TOS	ND	Anterior	Supra and infraclavicular incisions
Anusitviwat et al. 2021 [3]	F	17	R	Pain on palpation	ND	Anterior	infraclavicular with resection of clavicle
Tvedten et al. 2021 [4]	F	5	R	Horner syndrome	1.8 × 1.8 × 1.6 cm	Entire	ND

TOS thoracic outlet syndrome

ND no data available

## Case presentation

**Fig. 1:**
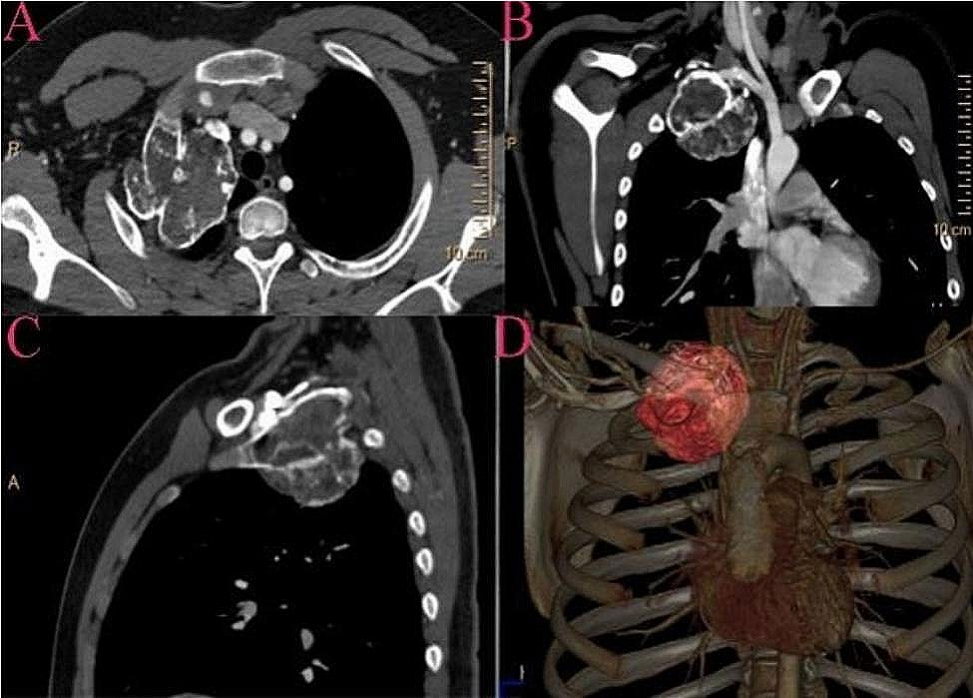
Chest computed tomography shows a bulging calcified mass arising from the right first rib. (**A**) Axial view. (**B**) Coronal view. (**C**) Sagittal view. (**D**) Chest 3D-CT images

**Fig. 2:**
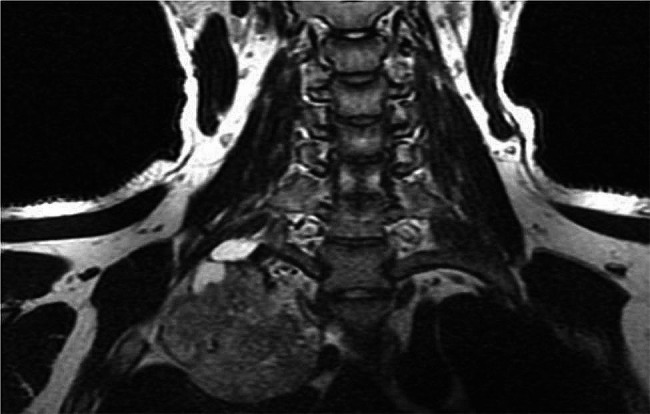
T2-weighted magnetic resonance images show a coronal view

An 18-year-old Chinese male presented with an incidental discovered raised calcified mass on the right first rib during a chest computed tomography (CT) scan (Fig. 1). The patient had no previous history of trauma or surgery and was in good health. The physical examination also revealed no abnormalities. The mass measured 6.9 × 6.4 × 6.7 cm (anterior/posterior transverse X cranial/caudal, respectively) and showed no soft tissue components. For further assessment, magnetic resonance imaging (MRI) was performed, which revealed a bright peripheral T2 signal of the right paraspinal mass indicative of a cartilaginous cap and good continuity of the medullary cavity. A careful visualization of the mass manifested that it was highly probably originated from the right first rib (Fig. 2). The finding was most consistent with osteochondroma.

**Fig. 3:**
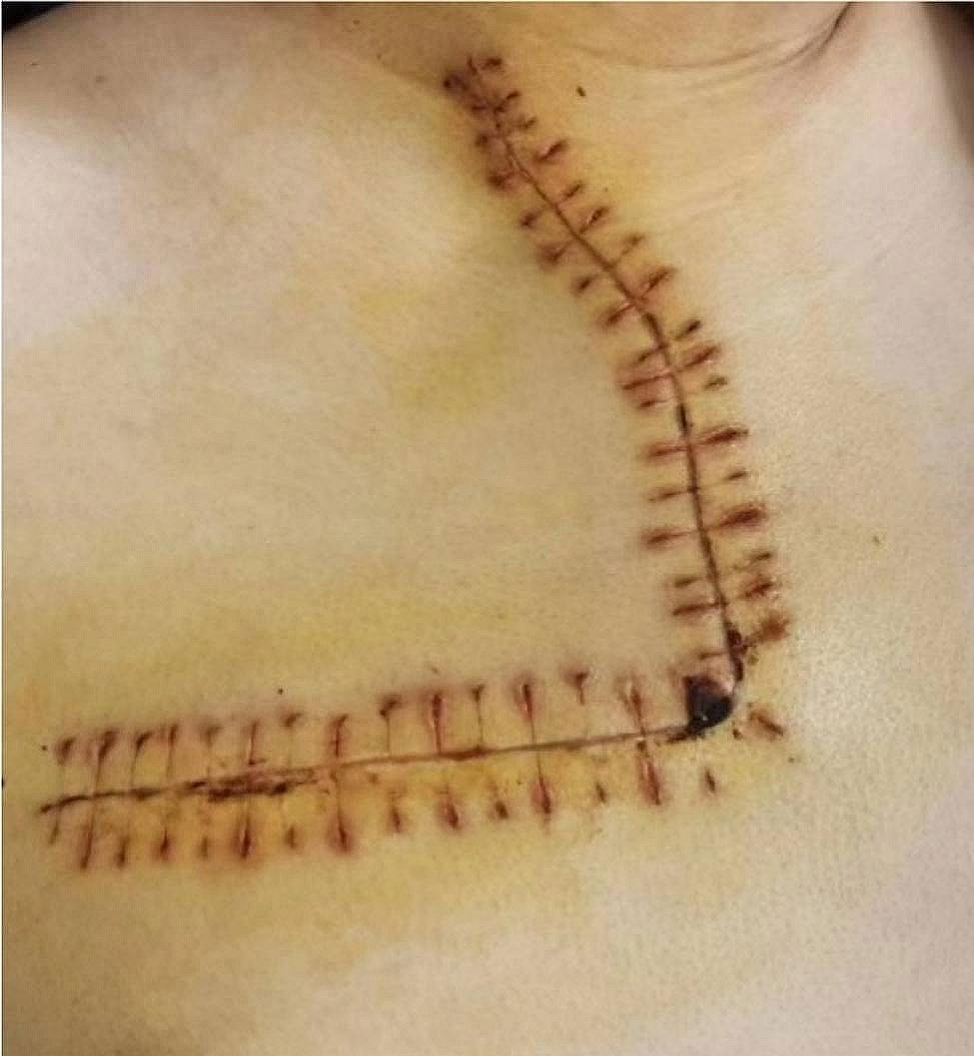
Inverse L-shaped incision in anterior approach

**Fig. 4:**
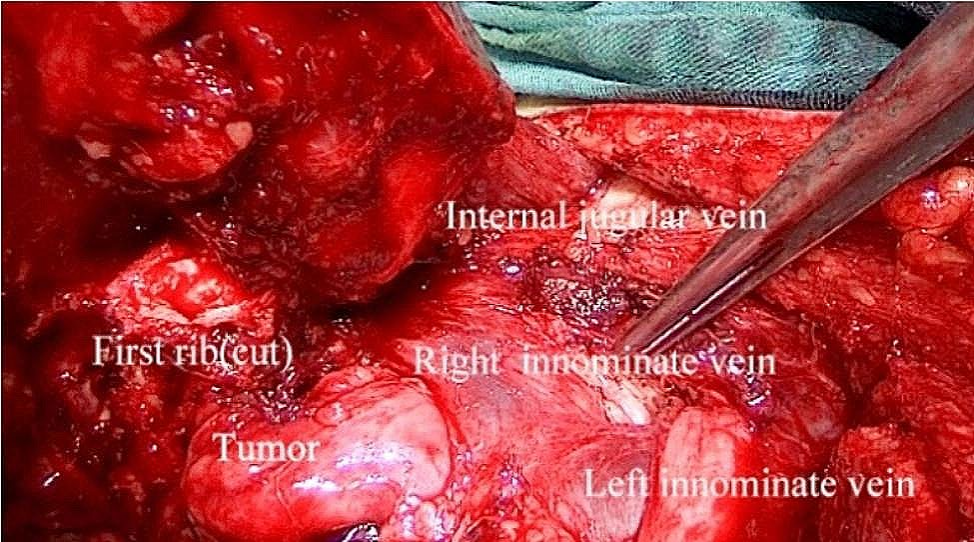
After turning over the clavicle and the free sternal stalk, the upper mediastinum and the important great vessels of the neck are clearly demonstrated

**Fig. 5:**
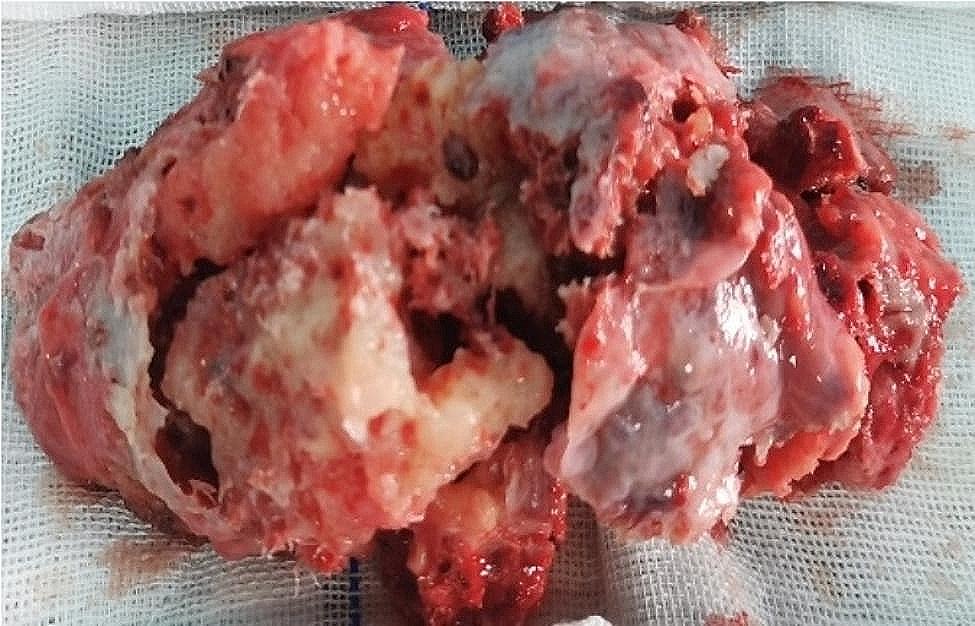
Specimen of the first rib with osteochondroma

**Fig. 6:**
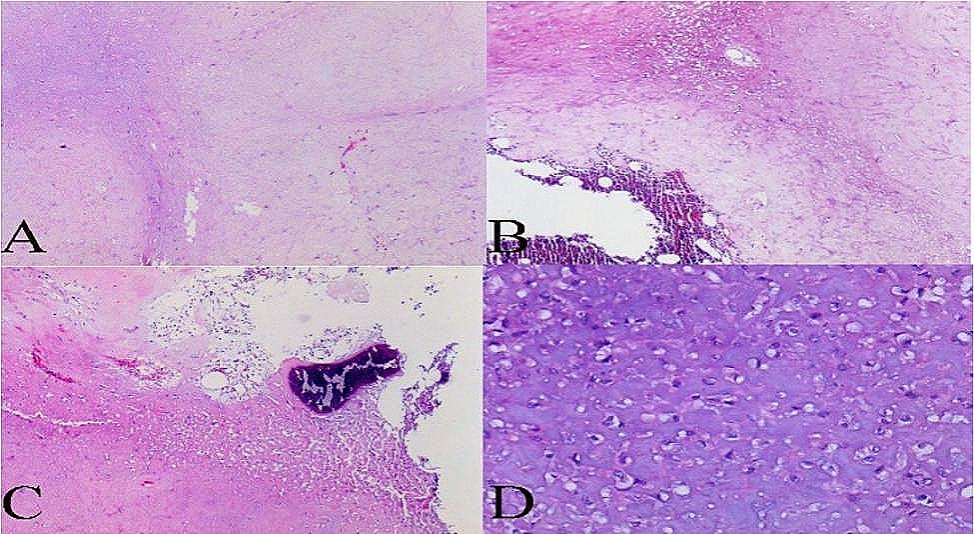
Histopathologic section of the resected specimen demonstrating osteochondroma. (**A**) The tumors were multinodular, partially fused, rich in cartilage matrix, and without atypia (Hematoxylin and eosin; ×40.). (**B**) The junction between the tumor and the normal bone marrow showed a pushing growth (Hematoxylin and eosin; X40.). (**C**) The tumor was focally sclerotic and calcified (Hematoxylin and eosin; ×40.). (**D**) The cytoplasm was vacuolated; the nuclei were small, with no nuclear fission (Hematoxylin and eosin; ×200.)

Intraoperatively, the patient was placed in a supine position under general anesthesia. The transmanubrial approach was used, and an inverse L-shaped skin incision was made (Fig. 3). The incision extended along the leading edge of the sternocleidomastoid muscle to the midpoint of the superior sternal fossa, descended from the anterior median line to the 2nd intercostal line, and continued outwards to the anterior axillary line. The subcutaneous tissue and muscle layers were separated, and the division of the internal mammary vessels was performed at the 2nd intercostal space. After the successive resection of the first costal cartilage and the costoclavicular ligament, the osteomuscular flap was uplifted. The brachiocephalic vein and subclavian vessels were identified, both of which were uninvaded by the tumor (Fig. 4). The first rib was removed intact (Fig. 5). The broken ends of the ribs were smoothed to avoid pricking the blood vessels and nerves. Bone wax was applied to stop the bleeding. The sternal stalk was fixed with wire suture and Nitinol grip plate suture. A chest tube was placed on the 7th intercostal midaxillary line. The incision was closed. The postoperative recovery was uneventful. The numbness on the ulnar side of the forearm was observed after the surgery, without motor dysfunction or Horner’s syndrome. Pathological examination confirmed the diagnosis of osteochondroma of bone (Fig. 6).

## Discussion and conclusions

Osteochondroma is a benign tumor of the skeletal system that rarely occurs in the ribs. Patients with rib osteochondroma were usually asymptomatic, but occasionally felt pain, and those with progression to thoracic outlet syndrome (TOS) were even more so [5, 6]. Three-dimensional reconstruction of computed tomography can determine the size, location, and internal structure of the tumor, as well as the adjacent relationship with the neighboring organs, playing a guiding role in the scope of surgical resection of the tumor. Histologically, osteochondroma is divided into three layers: fibrous membrane, cartilaginous cap, and bone. The outermost layer is a thin fibrous membrane that continues with the periosteum of the basal bone. The middle layer is the cartilaginous cap, which is usually less than 2 cm thick. If the thickness is > 2 cm and the shape is irregular, the possibility of malignancy should be considered [7]. Surgery is the main treatment for osteochondroma, highlighting the importance of early detection and intervention to prevent tumor enlargement, functional impairment, and malignant degeneration.

The upper part of the first rib is the subclavian artery, vein, and brachial plexus nerve. The lower part is the thoracic roof. The clavicle shields the front, and the back is covered by the scapula. The inside and the sternostalk form the sternocostal joint, and the deep part is the lung tip. Therefore, the deep anatomical position of the first rib leads to its difficult exposure, leaving the surgical resection of the first rib tumor a difficult problem for thoracic surgeons. Importantly, surgeons must carefully select the proper surgical approach and determine how to expose the lesions while protecting the peripheral vital vessels and nerves. The methods for excising the first rib have been widely reported in the literature, including the subaxillary arc incision approach, supraclavicular incision approach, subclavian incision approach, combined cervicothoracic approach, endoscopic subaxillary incision approach and video-assisted thoracoscopic surgery [8, 9].

The patient underwent a first osteochondroma resection and thoracic reconstruction through an inverse L-shaped incision in the anterior chest and a longitudinal split of the thoracic bone. This approach provided good surgical exposure and sufficient operating space for complete resection of the tumor and peripheral vascular neuroprotection. The mass of the first rib was removed under direct vision to avoid massive bleeding induced by damage to the surrounding great blood vessels due to unclear surgical field and anatomy during free tumor resection. The patient had symptoms of brachial plexus injury after surgery, with numbness on the ulnar side of the forearm. The patient recovered gradually after 2 months with no residual symptoms. It is important to note that nerve injury symptoms can be attributed to excessive intraoperative stretching during tumor exposure. Therefore, gentle manipulation during exposure of the subclavian tissue, avoidance of excessive stretching, and limited use of electrocoagulation are recommended.

In summary, the authors suggested that an inverse L-shaped incision in the anterior chest approach is necessary for the complete excision of first rib tumors. This surgical technique provides effective exposure of the diseased tissue under direct vision while minimizing the risk of subclavian arteriovenous and nerve injury. However, due to the rarity of bone tumors at this site and the limited number of patients in this study, larger sample sizes and multi-center studies are warranted.

## Data Availability

No datasets were generated or analysed during the current study.

## Abbreviations

CT: Computed Tomography
MRI: Magnetic Resonance imaging
TOS: Thoracic outlet syndrome
